# 

**Published:** 1892-10-29

**Authors:** 


### CONTENTS.

| | PAGE |
|---|---|
| **Vivisection: An Appeal to Reason** | 65 |
| **Passing Topics.**—Rates *versus* Subscriptions | 66 |
| **The Fruits of Vivisection.** By SIR ANDREW CLARK, Bart., M.D., F.R.S. | 67 |
| **Medical History from the Earliest Times.** By E. T. WITHINGTON, M.A., M.B., Oxon. XXIX.—Arabic Medicine—(2) The Eastern Caliphate | 68 |
| **Everybody's Page** | 69 |
| **The Attitude of Great Writers Towards Disease, XIV.**—Sir Thomas Browne | 70 |
| **Annotations.**—The Methods and Morals of Somerset House—The "Practical Man"—Why is THE HOSPITAL THE HOSPITAL? | 71 |
| **Notes and News** | 72 |
| **Editor's Letter-Box.**—Assistants' Manners and Nurses' Fees | 76 |
| **The Hospital Clinic—** | |
| ROYAL INFIRMARY, EDINBURGH—The Treatment of Flat Foot. | 73 |
| GLASGOW WESTERN INFIRMARY.—Varicose Veins | 73 |
| NEWCASTLE-UPON TYNE INFIRMARY.—On Amputation | 74 |
| **The Practioners Mirror and Retrospect—** | |
| SURGERY—The Treatment of Fractured Patella | 76 |
| **The Institutional Workshop—** | |
| WITHIN THE HOSPITALS.—Leeds General Infirmary | 77 |
| PRACTICAL DEPARTMENTS.—Children's Cots | 79 |
| HOSPITAL ADMINISTRATION.—The Royal Frederick's Hospital, Copenhagen | 80 |
| **Books Received** | 80 |
| **Scraps and Gleanings** | 80 |
| **The Student of Medicine.**—Vacancies | |

### EXTRA SUPPLEMENT.—THE NURSING MIRROR.

| | |
|---|---|
| **En Passant.**—Lincoln Diocesan Conference—St. Luke's Home, Vancouver—Foundlings and Fevers—American Jottings—Jedburgh District Nursing Association—Where to Go | xxv |
| **Lectures for Asylum Attendants.** By WILLIAM HARDING, M.B.—V. Food (*continued*) | xxvi |
| **Royal British Nurses' Association** | xxvi |
| **The Training of Male Nurses** | xxvii |
| **Appointments** | xxvii |
| **Notes and Queries** | xxvii |
| **Some Australian Experiences** | xxviii |
| **Everybody's Opinion.**—A Pleasant Lecture | xxix |
| **Presentations** | xxix |
| **The Dundee Nurses** | xxix |
| **For Reading to the Sick.**—The Sympathy of Christ | xxix |
| **Wants and Work rs** | xxix |
| **Off Duty.**—"The Result of a Chill." | xxx |

